# Contribution of the alternative model for DSM-5 personality disorders to relationship satisfaction

**DOI:** 10.3389/fpsyt.2023.1291226

**Published:** 2024-01-12

**Authors:** Claudia Savard, Mélissa Deschênes, Élodie Gagné-Pomerleau, Maude Payant, Kristel Mayrand, Marie-Chloé Nolin, Louis-Alexandre Marcoux, Dominick Gamache

**Affiliations:** ^1^Department of Educational Fundamentals and Practices, Université Laval, Québec, QC, Canada; ^2^CERVO Brain Research Centre, Québec, QC, Canada; ^3^Interdisciplinary Research Center on Intimate Relationship Problems and Sexual Abuse, Montréal, QC, Canada; ^4^School of Psychology, Université Laval, Québec, QC, Canada; ^5^Department of Psychology, Université du Québec à Montréal, Montréal, QC, Canada; ^6^Department of Humanities, Saint-Anne University, Pointe-de-l'Église, NS, Canada; ^7^Department of Psychology, Université du Québec à Trois-Rivières, Trois-Rivières, QC, Canada; ^8^Centre de Psychologie Québec, Québec, QC, Canada

**Keywords:** Alternative Model for Personality Disorders, relationship satisfaction, personality disorders, private practice clinics, intimate relationships

## Abstract

**Introduction:**

Personality is a central factor associated with relationship discord, conflicts, and separation, as well as with dyadic adjustment and relationship stability. The Alternative Model for Personality Disorders (AMPD) of the DSM-5 offers a hybrid model for understanding personality based on personality dysfunction (Criterion A) and pathological domains and facets (Criterion B). So far, few studies have integrated this model into the understanding of relationship quality. Therefore, the aim of this study was to examine the contribution of Criterion B to relationship satisfaction in individuals involved in an intimate relationship. We also explored the joint contribution of Criteria A and B, as well as their interaction effects, to relationship satisfaction.

**Methods:**

Participants were drawn from two clinical samples: patients with personality disorders (PD; *N* = 101) and clients consulting in private practice clinics (PPC; *N* = 350). They completed self-report questionnaires assessing relationship satisfaction and AMPD Criteria A (only for PPC sample) and B.

**Results:**

Hierarchical regressions showed that, for the PD sample, the Detachment and Negative Affectivity domains, especially the pathological facets of Intimacy Avoidance and Separation Insecurity, explained 22.5% of relationship satisfaction’s variance. For PPC clients, Detachment, Negative Affectivity, and Antagonism domains, and especially the pathological facets of Intimacy Avoidance, Anxiousness, and Grandiosity, contribute significantly to relationship satisfaction, explaining 14.8% of its variance. Criterion A elements did not evince incremental value to the regression models in the PPC sample, and no Criteria A and B interaction effects were found. Clinical implications as well as limitations of the study are discussed.

## Introduction

Many studies have documented personality as one of the most important variables in intimate relationships [i.e., interpersonal relationships that involves sexual and/or emotional intimacy (1)], most of them based on well-known models of personality [e.g., Big Five, (2)], and others on specific personality traits (3). Indeed, personality traits can impede the development, quality, and stability of intimate relationships in adulthood (1). Neuroticism (i.e., emotional instability and depression, self-doubt, anger, and hostility) from the Big Five personality model has been extensively linked with relationship discord, conflicts, intimate partner violence, and union dissolution (4, 5). Notably, this association has been observed both at high levels of Neuroticism and at very low scores, suggesting a curvilinear relationship between Neuroticism and relationship dissatisfaction (6). Moreover, other specific maladaptive personality traits such as psychopathy and perfectionism have also been identified as potential factors contributing to relationship dissatisfaction (7, 8). Conversely, other personality traits, like Conscientiousness, in both partners have been associated with greater relationship satisfaction (9). Specific processes described by Roberts et al. (10) demonstrate how personality traits can impede the quality of such relationships. For instance, personality plays a role in determining the extent to which individuals are exposed to various relationship events, such as frequency of conflicts. Additionally, it affects how individuals respond to their partner’s behaviors, while also eliciting certain behaviors from the partner in return.

The link between personality and love relationships has prompted researchers to study intimate relationships in individuals with personality disorders (PD). PDs are characterized by long-standing patterns of maladaptive thoughts, emotions, and behaviors that significantly impact an individual’s ability to engage in healthy and fulfilling relationships (11). Indeed, PDs are often thought of as relational disorders (12), that may manifest in various ways, such as struggles with vulnerability and intimacy in interpersonal relationships (13). Additionally, PDs are inherently connected to one’s sense of self, as individuals with these disorders often experience a lack of clarity or a distorted sense of their own identity (14). Considering the core nature of PDs, couples in which one or both partners have PDs often experience compromised functioning and conflicts (15), notwithstanding the fact that they tend to form unions together (15–17). Indeed, research has shown that PDs can exert a pervasive influence on various aspects of couple functioning, including communication, intimacy, conflict resolution, and overall relationship satisfaction (18, 19). For instance, individuals with borderline personality disorder (BPD) commonly exhibit impulsive behaviors, emotional instability, and intense fear of abandonment, leading to frequent relationship conflicts and difficulties in establishing and maintaining trust (16). Similarly, individuals with narcissistic personality disorder may show compromised empathic functioning and display an excessive need for admiration, which can create challenges in establishing reciprocal emotional connections and intimacy (20, 21). It is important to note that the presence of a PD affects not only the individual’s own relationship satisfaction but also their partner’s (22, 23). Furthermore, PDs and PD features have consistently been associated with intimate partner violence (both victimization and perpetration), with stronger associations found with borderline and antisocial PD (16, 24).

However, currently available data on PDs are primarily based on the traditional categorical approach outlined in the American Psychiatric Association’s *Diagnostic and Statistical Manual of Mental Disorders* [DSM-5 (11)]. The categorical approach defines PDs based on the presence of a certain number of diagnostic criteria and posits that PDs are discrete disorders. However, over the years, concerns have been raised regarding the limitations of this approach [e.g., poor reliability, high comorbidity, excessive reliance on the “PD not otherwise specified” category, arbitrary thresholds, limited clinical utility (25–27)].

In response to these concerns, the Alternative Model for DSM-5 Personality Disorders (AMPD) was introduced in 2013 in Section III (Emerging Measures and Models) of the DSM-5 (11). It offers a hybrid perspective on personality pathology that, firstly, focuses on impairments in personality functioning (Criterion A), which comprises Self and Interpersonal functioning, further broken down into four elements: Identity, Self-direction, Empathy, and Intimacy. The AMPD also highlights the central role of 25 pathological personality facets in the expression of personality pathology (Criterion B), categorized into five broad domains: Negative Affectivity, Detachment, Antagonism, Disinhibition, and Psychoticism.

Previous studies examining the relationship between marital satisfaction and the AMPD have consistently demonstrated strong associations between maladaptive domains and facets and relationship dissatisfaction in community couples, both at baseline and in follow-up assessments (28–30). Specifically, the Negative Affectivity, the Detachment, and—although to a lesser extent—the Antagonism domains have consistently shown a significant negative association with relationship satisfaction. An individual’s higher score on those domains not only affects one’s own relationship satisfaction negatively but also has a detrimental impact on their partner’s (28, 30). Furthermore, Sexton et al. (31) have highlighted the potential interaction effects between AMPD Criterion A and B (e.g., low Antagonism predicts higher relationship satisfaction but only when Empathy is not impaired).

### Objectives and hypotheses

Despite the promising findings on the associations between personality and marital satisfaction, the small sample sizes and their composition (i.e., mostly community participants) limit their generalizability. Additionally, previous studies only used domain or composite scores of AMPD categorical PD (antisocial, borderline, avoidant, narcissistic, obsessive-compulsive, and schizotypal) in the study of relationship satisfaction, without exploring Criterion B facets specifically (28–30). Finally, only one study incorporated both AMPD Criteria A and B (31). Therefore, the current study aimed to investigate the associations between maladaptive personality domains and facets from the AMPD, and relationship satisfaction in two clinical samples: one including patients with a PD, and another comprising clients in private practice clinics. The associations between personality functioning and the potential interaction effects between AMPD Criteria A elements and B domains on relationship satisfaction were also explored in the PPC sample.

Based on previous research findings, we hypothesized that the AMPD Criterion B domains of Detachment (specifically the Intimacy avoidance facet), Negative Affectivity, and Antagonism, would contribute to lower relationship satisfaction. Considering the limited empirical support concerning Criterion A associations with relationship satisfaction as well as for interaction effects between Criterion A and B, we did not have specific hypotheses and rather opted for an exploratory approach by testing all possible combinations.

## Methods

### Participants

Two clinical samples were used in this study. First, 101 patients (*M*_age_ = 32.76, *SD* = 9.33; 81.2% women) in an intimate relationship and consulting in a day hospital treatment program for PD following a crisis episode agreed to complete questionnaires, in person, at the beginning of their treatment program. Participants were informed that the data would only be used for research purposes and that no compensation was offered. Most participants had a diagnosis (retrieved from patient files) of BPD or borderline traits (77.5%), while 8.2% had a narcissistic PD diagnosis, 4.1% had dependent PD, and 10.1% had unspecified or mixed PD. Comorbid diagnoses were also present in 66.3% of the sample, the most frequent being substance-related and addictive disorders (22.4%), adjustment disorder (10.2%), and attention-deficit/hyperactivity disorder (9.2%). Regarding annual income, 86% of the sample earn less than CAD 55,000$ and 27.3% have at least one child. They were married or engaged in a cohabiting or non-cohabiting relationship in 65% of the cases; 6% were recently or temporarily separated, and 29% were seeing someone but considered their relationship not serious enough or too recent to categorize themselves as “in a relationship”. Those participants were instructed to refer to their ex or their actual date as their intimate partner. Relationship duration ranged from 2 months to 24 years (*M* = 6.48, SD = 6.59).

Second, a sample of 350 clients engaged in an intimate relationship and consulting for individual psychotherapy in private practice clinics (PPC; *M*_age_ = 35.44, SD = 9.75; 62.3% women) agreed to complete questionnaires at intake. No formal diagnosis was available for this sample. Regarding annual income, 53.2% of the sample earned CAD 55,000$ or more, and 54.1% had at least one child. All participants were married, engaged in a cohabiting or non-cohabiting relationship. Relationship duration ranged from 1 month to 45 years, with a mean duration of 8.87 years (SD = 7.59).

### Instruments

#### Relationship satisfaction

The validated 4-item French version of the *Dyadic Adjustment Scale* [DAS-4 (32)] is a self-report questionnaire used as a continuous measure of relationship satisfaction, with higher scores indicating greater satisfaction. In the current study, it showed good internal consistency [Cronbach alphas (α) respectively 0.89 and 0.78 for the PPC and PD samples].

#### Pathological personality traits

The Personality Inventory for DSM-5 Faceted Brief Form [PID-5-FBF (33)] is a widely used 100-item self-report instrument to evaluate the 25 facets of Criterion B, derived from its five higher-order domains: Negative Affectivity, Detachment, Antagonism, Disinhibition, and Psychoticism. Items are rated on a four-point Likert scale from 0 (*Very false or often false*) to 3 (*Very true or often true*), with a higher score representing higher levels of maladaptive traits. The French adaptation shows promising psychometric properties and invariance across language and gender (34, 35). In both samples, internal consistency ranged from α = 0.85 (Psychoticism) to α = 0.89 (Negative affectivity) for domains. In the PD sample, internal consistency for facets ranged from α = 0.64 (Irresponsibility) to α = 0.92 (Attention seeking), and from α = 0.63 (Irresponsibility) to α = 0.92 (Distractibility) in the PPC sample.

#### Personality functioning (used in the PPC sample only)

The validated French version of the Self and Interpersonal Functioning Scale [SIFS (36)], is a 24-item self-report measure of personality functioning according to Criterion A of the AMPD formulation. It assesses four elements: Identity (α = 0.73), Self-direction (α = 0.69), Empathy (α = 0.71), and Intimacy (α = 0.74). A global score of personality functioning can also be computed (α = 0.87). Items are scored on a five-point Likert scale, ranging from 0 (*This does not describe me at all*) to 4 (*This describes me totally*). A higher score reflects greater impairment.

## Results

Variables were normally distributed (37, 38), and no outliers were detected. Based on non-parametric group comparisons^1^ (Mann–Whitney) on Criterion B domains and facets, as well as relationship satisfaction variable, participants from the PD sample showed higher scores on most maladaptive facets (except for Grandiosity and Manipulativeness), lower scores on relationship satisfaction, and significantly shorter relationship duration compared to the PPC sample (Table 1). Correlations between variables are showed in Supplementary Table S1; the correlation between relationship satisfaction and duration was significant for the PPC sample (*r* = 0.17, *p* = 0.002) but not for the PD sample (*r* = 0.12, *p* = 0.343). Consequently, two sets of hierarchical linear regression models were computed separately for each sample, one using the domains as predictors and the other using facets. For the PPC sample, relationship duration was entered in a first step as a control variable. Domains (or facets) were then entered using a stepwise method. A third and a fourth model were also tested for the PPC sample, entering relationship duration at step one, personality functioning elements at step 2, and personality domains (or facets) at step 3. A more stringent significance level of 0.002 was determined based on GPower (39) to detect a large effect size (Cohen’s *f*^2^ = 0.35) with a statistical power of 0.80 to partially alleviate the inflated risk of type I error due to the high number of predictors and analyses.

**Table 1:** Descriptive statistics and group comparisons for personality disorder and private practice clinics samples.

Variables	PD Sample (*n* = 101)		PPC Sample (*n* = 350)		*U*	*d*
	*M*	*SD*	*M*	*SD*		
Relationship satisfaction	11.54	3.96	13.39	3.76	2204.00^***^	−0.49
Relationship duration	6.48	6.59	8.87	7.59	14837.50^***^	−0.33
**AMPD Criterion A**						
Identity			1.57	0.78		
Self-direction			1.22	0.71		
Empathy			0.77	0.61		
Intimacy			0.80	0.64		
Total score			1.09	0.53		
**AMPD Criterion B**						
Negative affectivity	1.97	0.62	1.20	0.64	6641.00^***^	0.64
Anxiousness	2.09	0.71	1.46	0.86	10006.00^***^	0.83
Depressivity	1.53	0.88	0.46	0.60	5490.00^***^	0.67
Emotional lability	2.10	0.71	1.13	0.74	6072.50^***^	0.74
Hostility	1.54	0.75	0.94	0.71	9507.50^***^	0.72
Perseveration	1.64	0.70	0.95	0.60	7853.50^***^	0.62
Separation insecurity	1.72	0.94	1.00	0.73	9490.00^***^	0.78
Submissiveness	1.65	0.70	1.28	0.70	12751.00^***^	0.71
Detachment	1.22	0.54	0.59	0.47	6670.00^***^	0.49
Anhedonia	1.60	0.83	0.73	0.70	7369.50^***^	0.73
Intimacy avoidance	0.78	0.71	0.35	0.43	11096.00^***^	0.50
Restricted affectivity	1.00	0.69	0.79	0.71	13648.00^**^	0.70
Suspiciousness	1.09	0.69	0.46	0.49	7883.50^***^	0.54
Withdrawal	1.25	0.71	0.68	0.63	9232.50^***^	0.65
Antagonism	0.63	0.52	0.47	0.44	13729.00^**^	0.46
Attention seeking	1.44	0.97	1.13	0.79	13733.00^**^	0.83
Callousness	0.43	0.67	0.26	0.45	14790.50^*^	0.51
Deceitfulness	0.71	0.68	0.41	0.49	12182.50^***^	0.53
Grandiosity	0.42	0.55	0.35	0.45	16117.00	0.47
Manipulation	0.78	0.75	0.66	0.59	15901.00	0.63
Disinhibition	1.54	0.54	0.88	0.57	6779.50^***^	0.56
Distractibility	1.98	0.76	1.31	0.88	9669.50^***^	0.86
Impulsivity	1.62	0.75	0.75	0.72	7124.50^***^	0.73
Irresponsibility	1.03	0.65	0.57	0.54	9572.00^***^	0.56
Rigid Perfectionism	1.64	0.82	1.17	0.75	11228.50^***^	0.76
Risk taking	1.13	0.79	0.68	0.66	11531.50^***^	0.69
Psychoticism	0.79	0.58	0.37	0.43	8643.00^***^	0.46
Eccentricity	1.27	0.81	0.60	0.72	8811.00^***^	0.74
Cognitive and perceptual dysregulation	0.50	0.67	0.22	0.39	12464.00^***^	0.46
Unusual beliefs and experiences	0.61	0.62	0.30	0.46	11057.50^***^	0.50

AMPD, Alternative Model for DSM-5 Personality Disorders. ^*^p < 0.05. ^**^p < 0.01. ^***^p < 0.001.

Results showed that for PD patients, the Detachment and Negative Affectivity domains explained 16.3% of relationship satisfaction’s variance (Table 2). More specifically, results based on facets indicated that Intimacy Avoidance (Detachment) and Separation Insecurity (Negative Affectivity) explained 22.5% of relationship satisfaction’s variance.

**Table 2:** Multiple linear regressions of relationship satisfaction with AMPD Criteria B personality variables for personality disorders patients and private practice clinics clients.

	B	ES B	β	*t*	*R* ^2^
**Personality disorders sample**					
AMPD Criterion B domains					0.163
Detachment	−2.11	0.70	−0.29	−2.99	
Negative affectivity	−1.56	0.62	−0.25	−2.53	
AMPD Criterion B facets					0.225
Separation insecurity	−1.61	0.38	−0.38	−4.19	
Intimacy avoidance	−1.97	0.51	−0.36	−3.90	
**Private practice clinics sample**					
AMPD Criterion B domains					0.100
Relationship duration	−0.05	0.00	−0.13	−2.50	
Negative affectivity	1.18	0.34	0.20	3.48	
Detachment	−1.58	0.44	−0.20	−3.56	
Antagonism	−1.64	0.45	−0.19	−3.66	
AMPD Criterion B facets					0.148
Relationship duration	−0.01	0.00	−0.13	−2.65	
Grandiosity	−1.95	0.42	−0.23	−4.63	
Anxiousness	0.95	0.24	0.22	3.93	
Intimacy avoidance	−1.57	0.49	−0.18	−3.21	

AMPD, Alternative Model for DSM-5 Personality Disorders. All paths are significant at *p* < 0.001.

For the PPC sample, Detachment, Negative Affectivity, but also Antagonism, explained 10% of relationship satisfaction’s variance when controlling for relationship duration. More specifically, Intimacy Avoidance (Detachment) and Grandiosity (Antagonism) were significant predictors of relationship satisfaction, explaining 14.8% of its variance. The model also identified Anxiousness as a statistically predictor of better relationship satisfaction.

Supplemental exploratory regression models were tested using jointly Criterion A elements and Criterion B domains (and facets) to predict relationship satisfaction in the PPC sample; Criterion A did not provide incremental value in the statistical prediction of relationship satisfaction (see Supplementary Table S2). Potential moderation effects of Criteria A (elements and total score) on Criterion B domains-relationship satisfaction associations previously identified as significant were also tested, using the extension Process 4.3 in SPSS v.29.0 (40). Contrary to Sexton et al.’s (31) procedure (who separated their sample based on adaptive and maladaptive personality functioning and traits), we used continuous scores for AMPD Criteria A and B, and for relationship satisfaction. Still, no results emerged as significant.

## Discussion

The aim of this study was to investigate the contribution of AMPD personality functioning and pathological personality domains and facets to relationship satisfaction in participants involved in an intimate relationship drawn from two clinical settings. Results indicate that multiple personality domains and pathological facets are associated with relationship satisfaction, with only Intimacy Avoidance (AMPD Criterion B) emerging as a significant statistical predictor across samples. This finding supported our hypothesis and is in line with previous studies reporting that individuals high on Detachment, but mostly on Intimacy Avoidance, tend to be less satisfied in their intimate relationship (28, 30). These results can be understood through the lens of attachment theory. Indeed, attachment avoidance is well known to be associated with relationship dissatisfaction, especially among men (41). People with avoidant attachment styles are independent, self-directed, and most of the time, uncomfortable with emotional intimacy and dependency. They tend to shy away from commitment and when they do engage in a relationship, they complain about feeling suffocated. Moreover, partners of individuals with avoidant attachment can become quite dissatisfied, which can translate into complaints, conflicts, and demand-withdrawal communication patterns (42), ultimately undermining satisfaction in the avoidant partner.

Separation insecurity (Negative Affectivity) was also associated with lower relationship satisfaction in the PD sample. This finding is in line with previous work showing that insecure attachment styles are predominant in individuals with PD, especially BPD [see (43) for a meta-analysis], which was overrepresented in our PD sample, and are known to be detrimental to intimate relationships (44–46). Indeed, previous studies showed that Detachment and Negative Affectivity are associated with attachment avoidance and anxiety, and with insecure attachment styles (46–48). In the present study, the joint presence of Criterion B Intimacy Avoidance (Detachment) and Separation Insecurity (Negative affectivity) in participants from the PD sample describes a disorganized (also referred to as Fearful-Avoidant^2^) attachment style that may result in frantic yet unsuccessful efforts to stave off separation and to keep distance from the partner (49). This result supported our hypotheses about the contribution of facets from the Detachment and Negative Affectivity domains in relationship dissatisfaction.

In the PPC sample, Grandiosity, which is included in the AMPD algorithm for diagnosing narcissistic personality disorder and describes an attitude of egocentrism and entitlement, also seems to predispose individuals to experience lower levels of relationship satisfaction. People with this trait may believe that they deserve better than their current relationship, regardless of their partner’s efforts. As a result, they may feel that their need for recognition is unfulfilled, leading to relationship dissatisfaction. This finding is in alignment with previous research that has consistently documented associations between narcissism and negative outcomes in romantic relationships, which include infidelity [e.g., (50)], communication problems (51), and intimate partner violence (52). However, the idea that an unquenchable need for recognition and attention can lead people with egocentric, condescending, and entitled attitudes to consistently feel dissatisfied in intimate relationships seems true only for clients consulting in private practice clinics but surprisingly not in PD patients, whose Grandiosity scores did not differ from PPC patients’. One possible explanation may be related to the composition of the PD sample, which included mostly women [known to score significantly lower than men on Grandiosity (34)] displaying borderline personality features, while Grandiosity is included in the narcissistic personality disorder algorithm (11). PD participants may also have underreported their pathological antagonistic traits due to a lack of insight or social desirability.

Surprisingly, Grandiosity was the only facet from the Antagonism domain that significantly contributed to relationship satisfaction, which only partially supports our prediction. One explanation could be that individuals with pronounced antagonistic traits are less likely to maintain intimate relationships and instead, engage in short-term, utilitarian relationships, as previously demonstrated (53). Additionally, individuals with antagonistic traits may be less inclined to seek consultation or therapy because they do not initially see any reason to change (54). Interestingly, previous studies have indicated that individuals with pathological personality traits or PDs tend to form relationships with similar partners (15–17). Therefore, it is plausible that two partners both with antagonistic traits could potentially experience greater satisfaction within their romantic relationship. This is what suggest the results from the study of Kardum et al. (55), who showed that dissimilarity, and not similarity, on Dark Triad traits is associated with low marital satisfaction. To further explore this hypothesis, future studies should consider using similarity coefficients and actor-partner models with AMPD Criteria A and B in couple samples.

Being worried about situations or uncertainty (Anxiousness from the Negative Affectivity domain) was found to be associated with better relationship satisfaction in individuals consulting for psychotherapy in private practice clinics. This counterintuitive result is at odds with some previous well-established findings, notably from studies showing that high levels of Neuroticism are associated with relationship dissatisfaction (4). Based on these findings, we hypothesize that at higher or clinical levels, anxiety could indeed have a negative impact on couple relationships and interactions. However, at subclinical levels, these characteristics may make individuals more sensitive or insecure when faced with relationship tensions, leading them to quickly resolve the situation. This explanation is in line with the curvilinear effect found for Neuroticism and relationship satisfaction (6). Individual with subclinical levels of anxiety may also find reassurance and support in their partner, who acts as a comforting figure, helping them overcoming their difficulties. However, further studies will be needed to confirm the presence of such a link or whether this is a result specific to this sample.

The absence of significant results concerning the contribution to AMPD Criterion A to relationship satisfaction in the PPC sample, as well as the absence of interaction effects between Criteria A and B, is somewhat surprising, considering the previous results reported by Sexton et al. (31). One explanation could be that the level of personality dysfunction in the PPC sample was quite low, with 69.1% of the sample displaying no PD or personality difficulty and only 6.3% reaching the threshold for moderate or severe PD according to the PD degrees of severity proposed by Gamache et al. (56). Data on personality functioning in the PD sample, which was unfortunately unavailable, could have shed additional light on the importance of personality functioning and its interaction with pathological personality facets on relationship satisfaction. The use of a 4-item version of the Dyadic Adjustment Scale could also have limited score variability. Given that there are still few data on the contribution of Criterion A to marital satisfaction, further studies seem necessary. Of note, previous research suggests that, with a few recent notable exceptions (57, 58), incremental validity of Criterion A over Criterion B remains limited for the prediction of outcome variables; some have suggested that it reflects redundancy and that Criterion A as currently defined is expendable (59) or that personality dysfunction self-reports are problematic (60), while others claim that it is rather Criterion B that is redundant and should be redefined since its domains and traits are saturated with dysfunction (61). The debate remains open regarding the optimal definition and interplay between Criteria A and B.

### Clinical considerations

Based on previous results, pathological facets pertaining to attachment avoidance and anxiety play a central role in relationship quality for PD patients. Therefore, therapeutic approaches known to have a specific impact on attachment should be prioritized (62). Among them, Emotionally Focused Therapy (63), Mentalization-Based Therapy (64), and Transference-Focused Psychotherapy (65, 66), which have all been empirically validated with patients suffering from PD, appear to be sound options. In helping PD patients to develop a more integrated perception of themselves and others, those approaches also aim to improve patients’ interpersonal function. Low relationship satisfaction in PPC clients seems explained by a more diversified constellation of traits. First, the Detachment component (Intimacy Avoidance) appears central, meaning that they may become distressed by a sense of estrangement from others (including their intimate partner). For these clients, social skills training may be beneficial in reducing Detachment traits (67), and potentially improving the quality of their intimate relationship. Grandiosity from the Antagonism domain also seems to contribute to a low relationship satisfaction. Even if no empirically informed treatment has been formally validated with narcissism (68), intervention focusing on developing empathic perspectives toward the partner could help clients to temper their excessive expectations of admiration. It can also help them to perceive the empathic concerns of others toward them (69) and hence to feel less “neglected” by their partner. In addition, psychoeducation about the function of emotions and skills work on emotion regulation could be helpful to master threatening or overwhelming emotional experiences (21), which impact interpersonal functioning.

### Limitations

Several limitations of the current study must be acknowledged, and as a result, its conclusions should not be overstated. First, all variables were obtained from self-report measures, which introduces the possibility of underreporting personality or relationship difficulties. Incorporating clinician or spouse ratings of personality traits could provide a more nuanced and accurate assessment of personality pathology. Second, the cross-sectional nature of the study does not allow any inference on the direction of the association between our variables and tempers the main conclusion that personality affects marital functioning. Indeed, a competing hypothesis could be that individuals experiencing relationship conflict or breakup may report higher personality dysfunction and pathological traits because of the level of distress experienced. A longitudinal research design would be beneficial to explore the causal associations between personality dysfunction and pathological facets and marital functioning. Third, even if the significance level of predictors was <0.001 and corresponds to a large effect size, we cannot rule out type I error. The replication of this study could further contribute to identify the strongest predictors of relationship satisfaction among AMPD Criterion A elements and Criterion B domains and facets. Fourth, the Criterion A questionnaire was only administered in the PPC sample, making it impossible to establish the contribution of AMPD Criteria A in predicting relationship satisfaction and marital functioning in the PD sample. Finally, both samples from this study had an unbalanced gender ratio, particularly the PD sample, which prevented gender-specific statistical analyses. Conducting such analyses would be necessary in future studies, as previous research has shown gender differences in domain scores (with women scoring higher on Negative Affectivity and men scoring higher on Antagonism [35]) and in the association between AMPD Criterion B and relationship functioning (e.g., 29). Furthermore, most participants (92.3%) self-identified as heterosexual, and the PD sample primarily consisted of individuals with borderline PD or traits. As a result, the generalizability of the findings may be limited. Future studies should aim to include Criterion A measures in diverse and larger samples, including individuals with different sexual orientations and identities and a wider range of PD diagnoses, to enhance the generalizability of the results.

## Conclusion

The present findings suggest that the AMPD model, especially Criterion B Negative Affectivity and Detachment domains provides valuable insights on relationship satisfaction. Apart from the Intimacy Avoidance facet, which is central to relationship satisfaction in diverse clinical samples, different pathological facets seem to be associated with in relationship dissatisfaction depending on the clientele. The associations observed in this study highlight the clinical significance of pathological personality domains and facets in understanding relationship satisfaction and overall well-being (28). Consequently, these manifestations of personality pathology could be considered as specific targets for personality-focused treatment interventions.

## Data availability statement

The datasets presented in this article are not readily available because of the local ethics committee restrictions. Requests to access the datasets should be directed to claudia.savard@fse.ulaval.ca.

## Ethics statement

The studies involving humans were approved by the Comité d’éthique à la recherche de l’Université Laval and by the Comité d’éthique à la recherche sectoriel en neurosciences et santé mentale of the Centre intégré universitaire de santé et de services sociaux de la Capitale-Nationale (CIUSSS-CN). The studies were conducted in accordance with the local legislation and institutional requirements. Written informed consent was obtained from all participants.

## Author contributions

CS: Conceptualization, Data curation, Formal analysis, Funding acquisition, Investigation, Methodology, Project administration, Resources, Writing – original draft, Writing – review & editing, Software, Supervision, Validation, Visualization. MD: Data curation, Writing – review & editing, Investigation, Software, Supervision. ÉG-P: Data curation, Writing – review & editing. MP: Data curation, Investigation, Writing – review & editing, Supervision. KM: Data curation, Funding acquisition, Investigation, Writing – review & editing, Software, Supervision. M-CN: Data curation, Investigation, Writing – review & editing, Software. L-AM: Resources, Writing – review & editing. DG: Funding acquisition, Investigation, Methodology, Writing – review & editing, Conceptualization.

## Footnotes

^1^Non-parametric group comparisons were used due to significant differences in variances between some variables and unequal sample sizes.

^2^Fearful-Avoidant attachment style describes people who do want intimacy and closeness, but at the same time, experience troubles trusting and depending on others due to their fear of getting hurt.
