# Supplementary material for: Contribution of the alternative model for DSM-5 personality disorders to relationship satisfaction
Source: Front Psychiatry. 2024 Jan 12;14:1291226. doi: 10.3389/fpsyt.2023.1291226 (PMC10811608; doi:10.3389/fpsyt.2023.1291226)
Supplement: Supplementary file 1 [file Table_1.docx]

**Supplemental material**

**Table S1.**

*Correlations between Criteria A and B Domains and Facets, Relationship Satisfaction, and Relationship Duration for Personality Disorders Patients and Private Practice Clinics Clients*

| Variables | Personality disorders sample (n = 101) | |  | Private practice clinics sample (n = 350) | | | | | |
| --- | --- | --- | --- | --- | --- | --- | --- | --- | --- |
|  | Relationship duration | Relationship Satisfaction |  | Relationship duration | Relationship Satisfaction | AMPD Criterion A | | | |
|  |  |  |  |  |  | Identity | Self-direction | Empathy | Intimacy |
| AMPD Criterion B Domains and Facets |  |  |  |  |  |  |  |  |  |
| Negative affectivity | -.17 | -.32*** |  | -.20*** | .12* | .61*** | .41*** | .25*** | .28*** |
| Emotional Lability | -.07 | -.15 |  | -.18*** | .08 | .58* | .39*** | .26*** | .24*** |
| Anxiousness | -.10 | -.25* |  | -.14* | .13* | .53*** | .35*** | .21*** | .27*** |
| Separation Insecurity | -.20 | -.33*** |  | -.18*** | .09 | .37*** | .28*** | .15*** | .19*** |
| Submissiveness | -.02 | -.12 |  | -.06 | .05 | .45*** | .34*** | .17*** | .26*** |
| Hostility | -.10 | -.13 |  | -.04 | -.10 | .40*** | .35*** | .45*** | .35*** |
| Perseveration | -.05 | -.36*** |  | -.10 | -.08 | .42*** | .44*** | .40*** | .31*** |
| Detachment | .09 | -.32*** |  | .02 | -.16** | .60*** | .41*** | .53*** | .63*** |
| Anhedonia | .09 | -.27** |  | -.02 | -.14** | .63*** | .40*** | .46*** | .46*** |
| Depressivity | .16 | -.29** |  | -.09 | -.02 | .70*** | .46*** | .36*** | .44*** |
| Intimacy Avoidance | -.06 | -.32** |  | -.03 | -.20*** | .26*** | .19*** | .31*** | .40*** |
| Suspiciousness | -.16 | -.24* |  | -.08 | -.11* | .47*** | .31*** | .44*** | .55*** |
| Withdrawal | .07 | -.15 |  | .05 | -.07 | .47*** | .34*** | .48*** | .63*** |
| Restricted Affectivity | -.03 | -.19 |  | .04 | -.20*** | .04 | .10 | .42*** | .29*** |
| Antagonism | -.15 | -.23* |  | -.06 | -.19*** | .08 | .28*** | .37*** | .28*** |
| Attention-Seeking | -.08 | -.10 |  | -.11* | -.05 | .05 | .24*** | .19*** | .09 |
| Callousness | -.07 | -.05 |  | -.09 | -.18*** | .13* | .17*** | .60*** | .43*** |
| Deceitfulness | .07 | -.20 |  | -.12* | -.11* | .19*** | .38*** | .36*** | .30*** |
| Grandiosity | -.08 | -.18 |  | -.01 | -.26*** | .02 | .17*** | .36*** | .25*** |
| Manipulativeness | -.27* | -.18 |  | -.02 | -.14** | .02 | .19*** | .25*** | .18*** |
| Disinhibition | -.16 | -.25* |  | -.18*** | .05 | .45*** | .64*** | .41*** | .32*** |
| Distractibility | -.16 | -.20* |  | -.17*** | .07 | .38*** | .42*** | .28*** | .23*** |
| Impulsivity | -.14 | -.13 |  | -.14* | .08 | .30*** | .60*** | .34*** | .26*** |
| Irresponsibility | -.12 | -.26** |  | -.11* | -.07 | .39*** | .53*** | .40*** | .31*** |
| Rigid Perfectionism (lack of) | .01 | -.28** |  | -.05 | -.08 | .31*** | .19*** | .30*** | .17** |
| Risk Taking | -.16 | -.07 |  | -.10 | -.15** | .04 | .23*** | .22*** | .13* |
| Psychoticism | -.24 | -.10 |  | -.11* | -.13* | .30*** | .31*** | .42*** | .32*** |
| Eccentricity | -.19 | -.11 |  | -.13* | -.13* | .30*** | .29*** | .45*** | .35*** |
| Cognitive and Perceptual Dysregulation | -.19 | -.12 |  | -.09 | -.08 | .28*** | .26*** | .32*** | .24*** |
| Unusual Beliefs and Experiences | -.18 | -.03 |  | -.04 | -.08 | .15** | .19*** | .19*** | .15* |

*Note. *p < 0.05. **p < 0.01. ***p < 0.001*

**Table S2.**

*Multiple Linear Regressions of Relationship Satisfaction with AMPD Criteria A and B Personality Variables for Private Practice Clinics Clients*

|  | B | ES B | β | *t* | *R*^2^ |  |
| --- | --- | --- | --- | --- | --- | --- |
| **Private practice clinics sample** | |  |  |  |  |  |
| AMPD Criterion A Elements and Criterion B Domains | | | |  | .100 |  |
| Relationship Duration | -0.01 | .00 | -.14 | -2.64 |  | |
| Negative Affectivity | 1.17 | .34 | .20 | 3.46 |  | |
| Detachment | -1.30 | .51 | -.17 | -2.58 |  | |
| Antagonism | -1.47 | .47 | -.17 | -3.10 |  | |
| AMPD Criterion A Elements and Criterion B Facets | | |  |  | .140 |  |
| Relationship duration | -0.01 | .00 | -.14 | -2.81 |  |  |
| Grandiosity | -1.78 | .45 | -.21 | -3.96 |  |  |
| Intimacy Avoidance | -1.81 | .47 | -.21 | -3.82 |  |  |
| Anxiousness | 0.80 | .23 | .18 | 3.50 |  |  |

*Note*. AMPD = Alternative Model for DSM-5 Personality Disorders.

All paths are significant at *p* < .001.
